# Perfusion Interval and Myocardial Injury in Minimally Invasive Mitral Valve Surgery: The Modifying Role of Aortic Cross-Clamp Duration

**DOI:** 10.1093/icvts/ivag174

**Published:** 2026-06-24

**Authors:** Chengying Shao, Wenshuai Mao, Lijun Guo, Zhiwei Liu, Xujie Hu, Jue He, Yong Cui, Zhibin Hu

**Affiliations:** Heart Center, Department of Cardiovascular Surgery, Zhejiang Provincial People’s Hospital (Affiliated People’s Hospital), Hangzhou Medical College, Hangzhou, Zhejiang, 310014, China; Heart Center, Department of Cardiovascular Surgery, Zhejiang Provincial People’s Hospital (Affiliated People’s Hospital), Hangzhou Medical College, Hangzhou, Zhejiang, 310014, China; Heart Center, Department of Cardiovascular Surgery, Zhejiang Provincial People’s Hospital (Affiliated People’s Hospital), Hangzhou Medical College, Hangzhou, Zhejiang, 310014, China; Heart Center, Department of Cardiovascular Surgery, Zhejiang Provincial People’s Hospital (Affiliated People’s Hospital), Hangzhou Medical College, Hangzhou, Zhejiang, 310014, China; Heart Center, Department of Cardiovascular Surgery, Zhejiang Provincial People’s Hospital (Affiliated People’s Hospital), Hangzhou Medical College, Hangzhou, Zhejiang, 310014, China; Heart Center, Department of Cardiovascular Surgery, Zhejiang Provincial People’s Hospital (Affiliated People’s Hospital), Hangzhou Medical College, Hangzhou, Zhejiang, 310014, China; Heart Center, Department of Cardiovascular Surgery, Zhejiang Provincial People’s Hospital (Affiliated People’s Hospital), Hangzhou Medical College, Hangzhou, Zhejiang, 310014, China; Heart Center, Department of Cardiovascular Surgery, Zhejiang Provincial People’s Hospital (Affiliated People’s Hospital), Hangzhou Medical College, Hangzhou, Zhejiang, 310014, China

**Keywords:** cardiopulmonary bypass, blood-enriched modified del Nido cardioplegia, myocardial protection, video-assisted cardiac surgery, minimally invasive mitral valve surgery

## Abstract

**Objectives:**

To assess how perfusion interval relates to myocardial injury and whether this is modified by aortic cross-clamp (ACC) duration in minimally invasive mitral valve surgery.

**Methods:**

We retrospectively analysed 556 patients receiving blood-enriched modified del Nido cardioplegia. The primary exposure and outcome were longest perfusion interval and base-10 logarithm-transformed peak cardiac troponin I (cTnI) within 48 hours. Analyses included multivariable regression, interaction and subgroup analysis, restricted cubic splines, sensitivity analysis, and logistic regression.

**Results:**

Longer perfusion intervals were initially associated with higher cTnI (regression coefficient 0.0097, 95% confidence interval [CI], 0.0035-0.0159; *P* = .002). This association disappeared after adjustment for factors including ACC time and number of perfusions (regression coefficient −0.0006, 95% CI, −0.0109 to 0.0097; *P* = .913). Aortic cross-clamp time remained independently associated with cTnI (regression coefficient 0.0099, 95% CI, 0.0013-0.0185; *P* = .024). In patients with prolonged ACC time, exploratory analysis suggested a non-linear increase beyond 80 minutes. In patients with ACC time of 90 minutes or less, perfusion interval was not clearly associated with peak cTnI. No significant associations were found with the composite of adverse events.

**Conclusions:**

The association between perfusion interval and myocardial injury depended on ACC duration. Single-dose blood-enriched modified del Nido cardioplegia appeared adequate when ACC time was 90 minutes or less. Longer intervals in prolonged procedures may matter, but the 80-minute threshold remains exploratory. These findings support a context-dependent myocardial protection strategy.

## Graphical abstract

**Figure:**
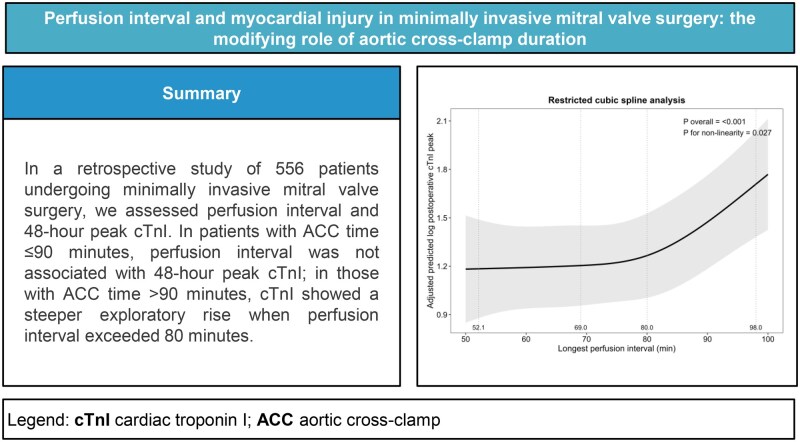

## INTRODUCTION

Mitral valve disease affects 1.9% of the global population, including stenosis and regurgitation forms.^1^ According to 2025 American Heart Association data, non-rheumatic degenerative mitral valve disease alone caused 40 000 deaths in 2021.^2^ Minimally invasive mitral valve surgery (MiMVS) through a right infra-axillary thoracotomy reduces trauma, hastens recovery,^3–5^ and is increasingly favoured.^6–8^ However, limited port access prolongs operative time and complicates uniform myocardial cooling, making myocardial protection more demanding than in sternotomy cases.^9^

Classic del Nido cardioplegia provides 60-90 minutes of arrest with one dose,^10–12^ but its low haematocrit may cause haemodilution.^13^ Myocardial protection is the cornerstone of successful cardiac surgery.^14^ We adopted a blood-enriched modified del Nido cardioplegia: a 4:1 mixture of the patient’s blood and the classic del Nido cardioplegia,^15^ which has shown safety in previous practice.^16^^,^^17^ But its optimal single-perfusion window remains undefined. Cardiac troponin I (cTnI) is a sensitive and specific marker of operative myocardial damage and is associated with prognosis.^18–20^ Because troponin release after cardiac surgery may continue for 48-72 hours, peak values during this period better reflect myocardial injury.^21^ Therefore, assessing the relationship between perfusion interval and postoperative cTnI is clinically relevant for refining myocardial protection and perioperative management.

With this goal, we designed a retrospective cohort study in patients undergoing MiMVS. By assessing the association between perfusion interval and 48-hour peak cTnI, and exploring its relationship with early postoperative outcomes, we aimed to define an evidence-based re-dosing window for blood-enriched modified del Nido cardioplegia.

## PATIENTS AND METHODS

### Study population

This single-centre retrospective observational study screened consecutive patients undergoing video-assisted right infra-axillary MiMVS with blood-enriched modified del Nido cardioplegia. To minimize the influence of procedural complexity on perfusion strategy and outcomes, the primary analysis was restricted to isolated mitral valve surgery. During the COVID-19 pandemic, surgery was postponed for patients with active or suspected infection until recovery and clinical clearance. Patients were excluded for concomitant procedures, including aortic or tricuspid valve surgery, coronary artery bypass grafting, congenital heart surgery or MAZE procedure, or unavailable key exposure or outcome variables.

### Data collection

All study data were obtained from the “Artificial Intelligence-Assisted Minimally Invasive Cardiovascular Surgery Full-Process Quality Management Platform” of the Department of Cardiovascular Surgery. Baseline variables included age, sex, body mass index, coronary artery disease, diabetes mellitus, hypertension, preoperative atrial fibrillation, New York Heart Association class, left ventricular ejection fraction, and preoperative cTnI. Intraoperative variables included cardiopulmonary bypass (CPB) time, aortic cross-clamp (ACC) time, number of perfusions, and blood transfusion.

### Ethical statement

This study was approved by the Ethics Committee of Zhejiang Provincial People’s Hospital (No. KY2024217) and followed the Helsinki Declaration. Written informed consent was waived due to the retrospective design. Data collection and storage for future use comply with the Declaration of Taipei, and ongoing use of data is monitored by the research ethics committee.

### Surgical procedure

All patients underwent endotracheal intubation under total intravenous anaesthesia. A video-assisted right infra-axillary MiMVS approach was used as previously described.^5^ Patients were placed in the supine position with the right hemithorax elevated by approximately 30°. A small incision was made along the right anterior axillary line, and the pleural cavity was entered through the fourth intercostal space. Through an atrial groove incision the valve was visualized and repaired or replaced under direct vision. Carbon dioxide insufflation was maintained throughout the intracardiac procedure.

### CPB and myocardial protection methods

Femoral arterial and venous cannulation was used to institute peripheral CPB. The circuit was primarily primed with Plasma-Lyte A and 20% albumin, and the patient was cooled to mild hypothermia. Throughout CPB we kept the haematocrit between 0.24 and 0.29, pumped at 2.0-2.4 l/(m^2^·min), and held mean arterial pressure in the 50-80 mmHg range. The solution was blood-enriched modified del Nido cardioplegia: a 4:1 mix of the patient’s own blood with the classic del Nido cardioplegia (**Table S1**).^16^ A single-use warm-blood cardioplegia set (Medtronic XP41B, USA) delivered the cardioplegia at 4°C. After the ACC was applied, an antegrade shot was given into the aortic root. The first dose was 4 parts blood to one part del Nido cardioplegia-A, pressurized to 200-250 mmHg, and run at 30 mL/kg up to a ceiling of 2 l. We planned to re-dose every 60 minutes with del Nido cardioplegia-B at half the initial volume.

### Exposure definition

Perfusion interval was defined from cardioplegia records as the time from the start of one del Nido infusion to the next, or to aortic unclamping if no further dose was given. The primary exposure was patient-level longest perfusion interval (LPI). Sensitivity metrics included mean interval and the number and proportion of intervals >80 or >90 minutes.

### Outcome measures

The primary outcome was postoperative peak cTnI within 48 hours, as a marker of perioperative myocardial injury. This window was selected because troponin release after cardiac surgery may be delayed, and the 48-hour peak better reflects cumulative injury than a single time-point measurement.^21^^,^^22^ Owing to its skewed distribution, cTnI was log_10_-transformed. The secondary outcome was a composite of adverse events, defined as the occurrence of any of the following: postoperative mortality, extracorporeal membrane oxygenation support, intra-aortic balloon pump support, continuous renal replacement therapy, permanent pacemaker implantation, or postoperative cerebrovascular events.

### Statistical analysis

Continuous variables are presented as median and interquartile range because most variables were skewed or had extreme values. Regression results are reported as regression coefficients or odds ratios, both with 95% confidence intervals (95% CIs). Group comparisons across LPI categories used the Kruskal-Wallis test for continuous variables and the χ^2^ or Fisher’s exact test for categorical variables. Associations between LPI and the primary outcome were assessed using multivariable linear regression, with log_10_-transformed peak cTnI within 48 hours as the dependent variable. A core model including prespecified demographic and clinical covariates was constructed, followed by additional adjustment for intraoperative factors (CPB time, ACC time, and number of perfusions). To further assess operative complexity, simplified models added ACC time and number of perfusions separately to the core model. Primary analyses used complete-case data to preserve observed values and the interpretation of intraoperative complexity. Sensitivity analyses assessed the effect of missing data using multiple imputation with chained equations and Rubin’s rules, and a missing-category approach in which missing New York Heart Association class was treated as “Unknown” while observed ejection fraction values were retained. Subgroup analyses were performed by ACC duration (≤90 vs >90 minutes), based on the recommended 60-90 minute interval for classical del Nido cardioplegia.^10–12^ Effect modification was tested using an LPI-by-ACC category interaction term. In patients with ACC time >90 minutes, restricted cubic spline assessed the dose-response relationship between LPI and log_10_-transformed postoperative peak cTnI within 48 hours. Sensitivity analyses used alternative interval-based metrics. For the secondary outcome, multivariable logistic regression was performed in both the overall cohort and the ACC time >90 minutes subgroup using selected LPI parameters.

All statistical tests were 2-sided, and *P* value < .05 was considered statistically significant. Analyses were performed using R Statistical Software (version 4.5.1).

## RESULTS

### Patient characteristics

**Figure 1.**
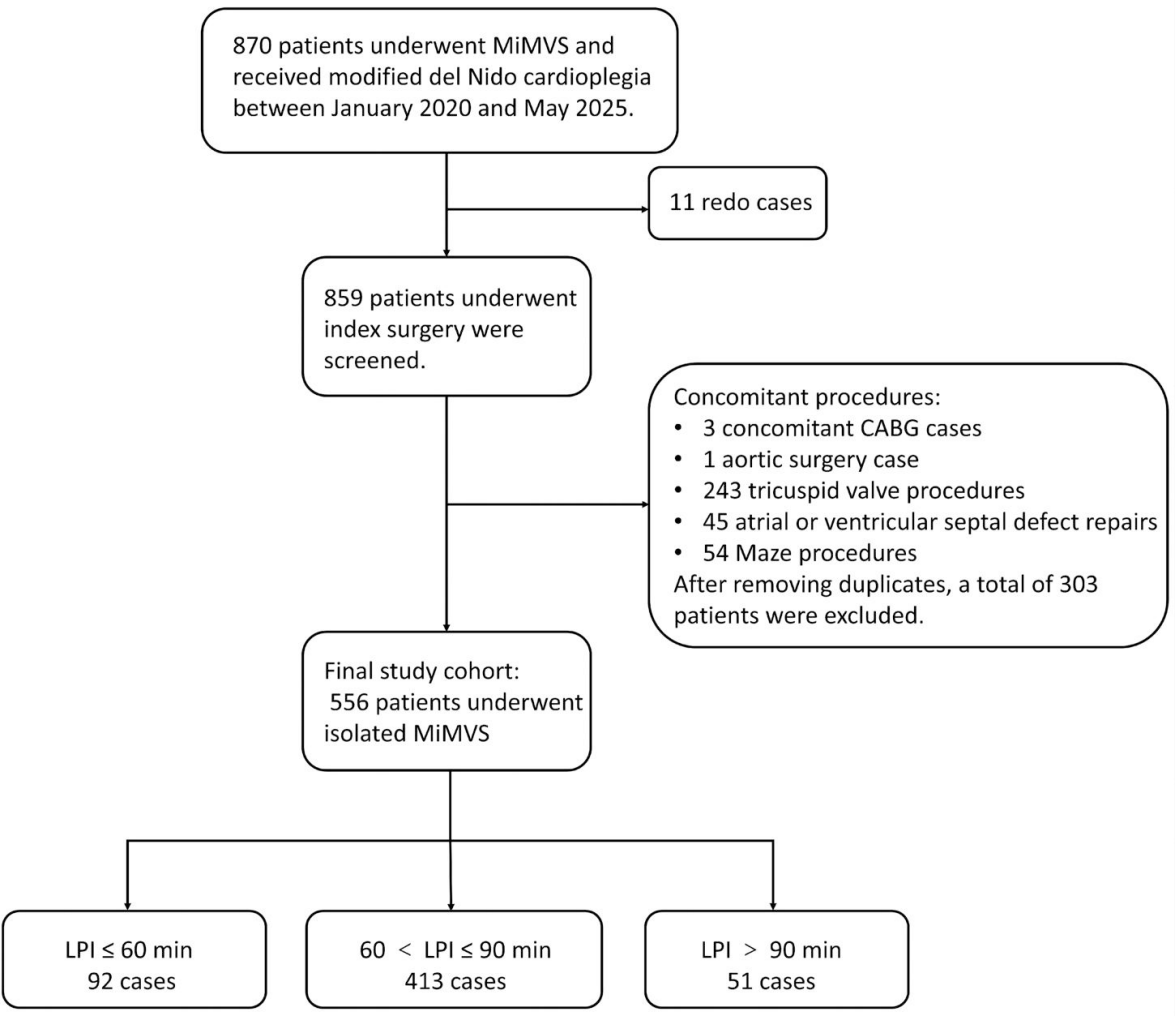
Patient Selection Flowchart. Abbreviations: LPI, longest perfusion interval; MiMVS, minimally invasive mitral valve surgery

A total of 556 patients were ultimately included in the final analysis after screening and applying the predefined exclusion criteria (**Figure 1**), including 291 mitral valve repair and 265 mitral valve replacement cases. Patients were divided into 3 groups according to the LPI: ≤60 minutes (*n* = 92), 60-90 minutes (*n* = 413), and >90 minutes (*n* = 51) (**Table 1**). Extent of missing data is presented in **Table S2**. Age increased across groups (median 54.0, 57.0, and 60.0 years; *P* = .045). Most baseline characteristics were similar among groups. Preoperative cTnI increased with longer perfusion interval (0.51, 0.69, and 1.04 μg/L; *P* = .001). Operative complexity also increased across groups, with longer CPB time, longer ACC time, different distributions of the number of perfusions, and progressive increases in maximum and mean perfusion interval.

### Postoperative peak cTnI within 48 hours across LPI groups

**Figure 2.**
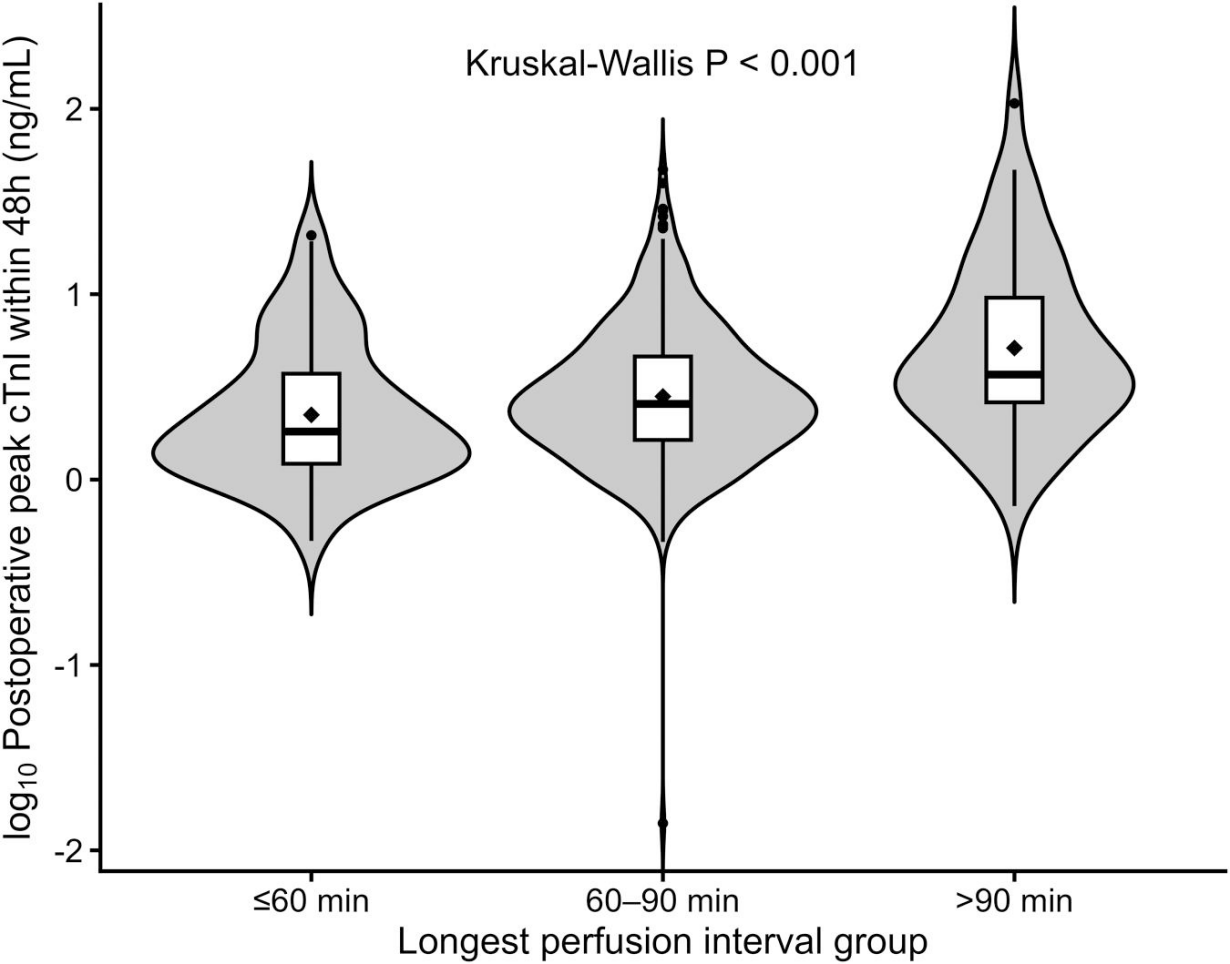
Distribution of Log_10_-Transformed Postoperative Peak cTnI Within 48 hours by LPI Groups. Violin plots with box plots are shown; diamonds indicate group means. Group differences were assessed using the Kruskal-Wallis test. Abbreviation: cTnI: cardiac troponin I

Postoperative peak cTnI within 48 hours differed across the 3 LPI groups on the log_10_-transformed scale (*P* < .001). The distribution shifted upwards with increasing LPI, with the >90 minute group showing the highest central tendency and the ≤60 minute group the lowest (**Figure 2**).

### Overall cohort analysis

In univariable analyses, ACC time, preoperative cTnI, CPB time, number of perfusions, LPI, blood transfusion, age, coronary artery disease, and hypertension were associated with higher log_10_-transformed postoperative peak cTnI within 48 hours (**Tables S3**). In the core clinical model, LPI remained associated with higher postoperative peak cTnI within 48 hours (β = 0.0097, 95% CI, 0.0035-0.0159; *P* = .002). After additional adjustment for intraoperative complexity variables, including CPB time, ACC time, and number of perfusions, this association was no longer significant (β = −0.0006, 95% CI, −0.0109 to 0.0097; *P* = .913). By contrast, ACC time remained significantly associated with postoperative peak cTnI within 48 hours (β = 0.0099, 95% CI, 0.0013-0.0185; *P* = .024). The adjusted *R*^2^ increased from 0.273 to 0.333 (**Table 2**; **Tables S4-S6**).

**Table 1.** Baseline and Intraoperative Characteristics by LPI Groups

Characteristic	*N*	≤60 minutes	60-90 minutes	>90 minutes	*P* value
		*N* = 92	*N* = 413	*N* = 51	
Age (years)	556	54.00 (41.50, 65.50)	57.00 (48.00, 67.00)	60.00 (52.00, 66.00)	.045
Male gender, *n* (%)	556	48 (52%)	229 (55%)	27 (53%)	.821
Body mass index (kg/m²)	556	23.75 (20.76, 25.12)	23.03 (21.22, 25.06)	23.87 (21.88, 26.22)	.317
Smoking status, *n* (%)	556	26 (28%)	106 (26%)	16 (31%)	.635
Alcohol consumption, *n* (%)	556	21 (23%)	100 (24%)	14 (27%)	.825
History of coronary artery disease, *n* (%)	556	17 (18%)	113 (27%)	12 (24%)	.198
History of diabetes mellitus, *n* (%)	556	9 (9.8%)	36 (8.7%)	6 (12%)	.758
History of hypertension, *n* (%)	556	30 (33%)	145 (35%)	22 (43%)	.435
History of cerebrovascular disease, *n* (%)	556	8 (8.7%)	37 (9.0%)	10 (20%)	.051
Preoperative atrial fibrillation, *n* (%)	556	14 (15%)	87 (21%)	14 (27%)	.208
Preoperative left ventricular ejection fraction (%)	541	64.00 (59.00, 68.00)	65.00 (60.00, 69.00)	65.00 (60.00, 69.00)	.156
New York Heart Association class	310				.313
1		0 (0%)	2 (0.9%)	0 (0%)	
2		26 (53%)	90 (40%)	19 (56%)	
3		16 (33%)	108 (48%)	11 (32%)	
4		7 (14%)	27 (12%)	4 (12%)	
Haemoglobin (g/L)	556	131.00 (118.00, 140.00)	129.00 (120.00, 142.00)	131.00 (119.00, 145.00)	.948
Albumin (g/L)	556	39.60 (36.90, 41.70)	38.80 (36.40, 41.30)	38.00 (34.70, 40.90)	.116
Alanine aminotransferase (U/L)	556	18.00 (11.00, 27.00)	18.00 (13.00, 30.00)	16.00 (12.00, 23.00)	.3
Aspartate aminotransferase (U/L)	551	21.00 (18.00, 28.00)	21.00 (17.00, 27.00)	16.00 (12.00, 23.00)	.6
Creatinine (μmol/L)	556	69.35 (61.25, 82.75)	72.10 (61.10, 85.30)	81.30 (63.60, 94.90)	.093
Lactate dehydrogenase (U/L)	551	181.00 (161.00, 214.00)	180.00 (159.00, 214.00)	194.00 (165.00, 216.00)	.3
Cardiac troponin I (μg/L)	556	0.51 (0.20, 0.98)	0.69 (0.24, 1.29)	1.04 (0.46, 3.05)	.001
Brain natriuretic peptide (pg/mL)	549	140.80 (97.30, 249.40)	160.20 (90.60, 240.20)	159.45 (101.30, 247.30)	>.9
Cardiopulmonary bypass time (minutes)	556	96.50 (87.00, 108.00)	127.00 (113.00, 153.00)	151.00 (137.00, 171.00)	<.001
Aortic cross-clamp time (minutes)	556	55.00 (51.00, 59.00)	82.00 (72.00, 105.00)	99.00 (93.00, 109.00)	<.001
Number of perfusions, *n* (%)	556				<.001
1		79 (86%)	279 (68%)	43 (84%)	
2		11 (12%)	123 (30%)	8 (16%)	
3		2 (2.2%)	11 (2.7%)	0 (0%)	
LPI (minutes)	556	55.00 (51.00, 58.00)	75.00 (68.00, 81.00)	98.00 (93.00, 102.00)	<.001
Mean perfusion interval (minutes)	556	53.00 (50.00, 56.25)	70.00 (62.00, 80.00)	98.00 (91.00, 101.00)	<.001
Parallel perfusion time (minutes)	556	18.00 (14.00, 23.00)	21.00 (16.00, 28.00)	24.00 (17.00, 30.00)	.003
Spontaneous rhythm recovery, *n* (%)	556	87 (95%)	375 (91%)	45 (88%)	.4
Rebeating time (minutes)	556	1.00 (1.00, 2.00)	2.00 (1.00, 3.00)	2.00 (1.00, 3.00)	.14
Blood transfusion, *n* (%)	556	19 (21%)	104 (25%)	14 (27%)	.6

Data are presented as median (interquartile range) or number (%). Comparisons used the Kruskal-Wallis test, χ^2^ test or Fisher’s exact test, as appropriate.

Abbreviation: LPI, longest perfusion interval.

**Table 2.** Selected Coefficients From Multivariable Linear Regression Models of LPI and Log_10_-Transformed Postoperative Peak cTnI Within 48 Hours

Variable	Model 1,	*P* value	Model 2,	*P* value
	β (95% CI)	(M1)	β (95% CI)	(M2)
LPI (min)	0.0097 (0.0035 to 0.0159)	.002	−0.0006 (−0.0109 to 0.0097)	.913
Age (years)	0.0050 (−0.0018 to 0.0119)	.150	0.0071 (0.0005 to 0.0137)	.035
Hypertension	0.2653 (0.0778 to 0.4528)	.006	0.1957 (0.0141 to 0.3773)	.035
Preoperative atrial fibrillation	−0.1846 (−0.4084 to 0.0392)	.106	−0.3185 (−0.5401 to −0.0970)	.005
Preoperative cTnI	0.1808 (0.1325 to 0.2292)	<.001	0.1469 (0.0987 to 0.1950)	<.001
Blood transfusion	0.3361 (0.1372 to 0.5349)	<.001	0.2498 (0.0552 to 0.4444)	.012
ACC time (min)	–	–	0.0099 (0.0013 to 0.0185)	.024
Number of perfusions	–	–	−0.1138 (−0.5184 to 0.2907)	.580
*N*	304		304	
Adjusted *R*²	0.273		0.333	

Complete-case analysis, *n* = 304. Data are β (95% CI). Model 1 was adjusted for demographic and clinical variables; model 2 was additionally adjusted for intraoperative factors (cardiopulmonary bypass time, ACC time, and number of perfusions).

Abbreviations: ACC, aortic cross-clamp; CI, confidence interval; cTnI, cardiac troponin I; LPI, longest perfusion interval.

To further assess this attenuation, simplified models were examined. Adding ACC time to the core clinical model markedly reduced the coefficient for LPI and abolished significance. Adding number of perfusions alone did not. When ACC time and number of perfusions were entered together, the association again disappeared (**Tables S7 and S8**). Results were consistent after multiple imputation (**Table S7**). Collinearity diagnostics showed greater overlap when ACC time and number of perfusions were modelled together (**Table S9**). These findings suggest that the attenuation of the association between LPI and postoperative myocardial injury was mainly driven by ACC time rather than by number of perfusions.

### Interaction with ACC duration

**Figure 3.**
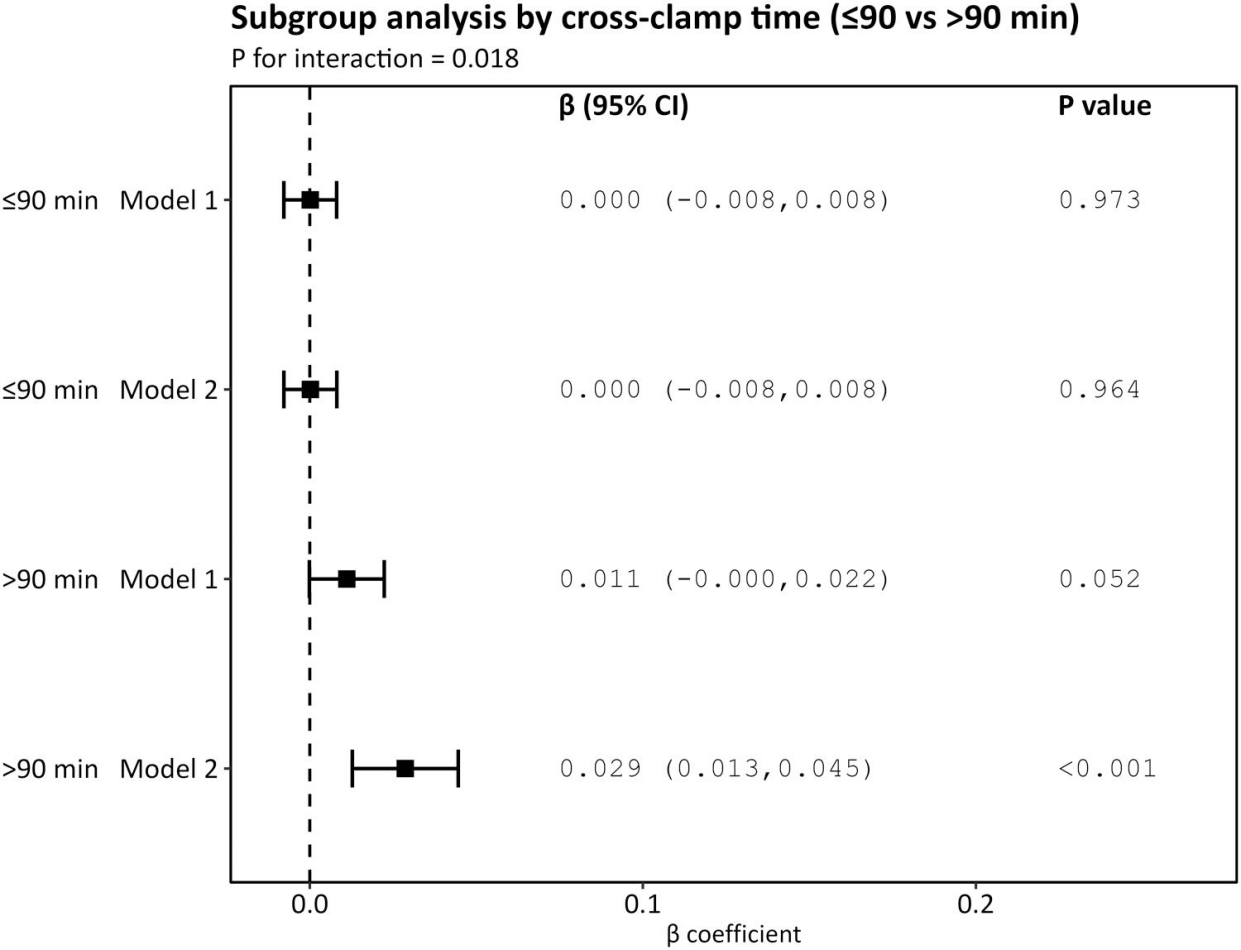
Subgroup Analysis of the Association Between Longest Perfusion Interval and Log_10_-Transformed Postoperative Peak cTnI Within 48 hours by Cross-Clamp Time (≤90 vs >90 Minutes). Forest plots show β (95% CI). Model 1 was adjusted for demographic and clinical covariates, and model 2 was additionally adjusted for number of perfusions. *P* for interaction was derived from the perfusion interval-by-cross-clamp time interaction term. Abbreviations: CI, confidence interval; cTnI, cardiac troponin I

We further evaluated whether the association between LPI and postoperative cTnI varied by ACC duration. In subgroup analyses, the association appeared stronger in patients with ACC time >90 minutes, whereas no clear association was observed in those with ACC time ≤90 minutes (**Figure 3**). This finding is compatible with adequate myocardial protection by a single perfusion within a 90-minute cross-clamp window, although the study was not designed as a direct comparison between single and repeated perfusion strategies. This pattern was consistent with the interaction model in the complete-case analysis, but the interaction was no longer statistically significant after multiple imputation (**Table S10**). Similarly, in the ACC time >90 minute subgroup, effect estimates remained positive but were not statistically significant in sensitivity analysis using both multiple imputation and the missing-category approach. Though the interaction between LPI and ACC duration was attenuated and not statistically significant, effect directions remained similar, supporting the robustness of the primary complete-case findings (**Table S11**).

### Analysis in patients with ACC time >90 minutes

**Figure 4.**
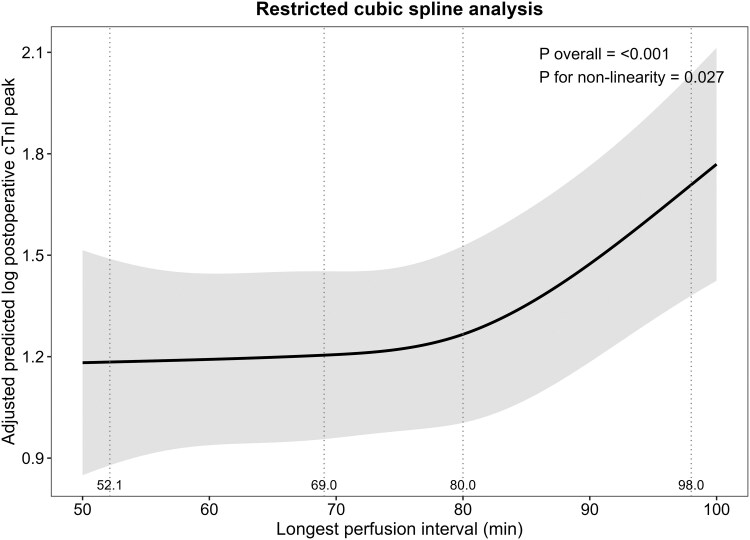
Restricted Cubic Spline Analysis of the Association Between Longest Perfusion Interval and Log_10_-Transformed Postoperative Peak cTnI Within 48 hours in Patients With Aortic Cross-Clamp Time >90 Minutes. The solid line shows adjusted predictions, and the shaded area indicates the 95% CI. The model was adjusted for demographic and clinical covariates and number of perfusions. Vertical dotted lines indicate knot locations. Overall and non-linear associations were tested by ANOVA. Abbreviations: CI, confidence interval; cTnI, cardiac troponin I

In patients with ACC time >90 minutes, restricted cubic spline analysis showed a significant overall association between LPI and log_10_-transformed postoperative peak cTnI within 48 hours (*P* < .001), with evidence of a non-linear relationship (*P* = .027) (**Figure 4**; **Table S12**). Using 4 knots located at 52.1, 69.0, 80.0, and 98.0 minutes, the adjusted spline curve remained relatively flat at lower exposure levels and then rose more steeply beyond approximately 80 minutes, suggesting a stronger increase in postoperative cTnI at longer perfusion intervals. Sensitivity analyses in this subgroup showed that mean perfusion interval was not associated with postoperative cTnI in Model 1, but became significant after additional adjustment for number of perfusions in Model 2 (β = .027, 95% CI, 0.013-0.042; *P* < .001) (**Table S13**). Other interval-based metrics, including the number of intervals >80 minutes, the proportion of intervals >80 minutes, the proportion of intervals >90 minutes, and the number of intervals >90 minutes, were not significantly associated with postoperative cTnI. No robust associations were observed between LPI parameters and the secondary outcome, either in the overall cohort or in the ACC time >90 minute subgroup (**Table S14**).

## DISCUSSION

In patients undergoing video-assisted right infra-axillary MiMVS with blood-enriched modified del Nido cardioplegia, longer perfusion intervals were initially associated with higher 48-hour peak cTnI, but this association disappeared after adjustment for intraoperative complexity, particularly ACC time, which remained independently associated. Subgroup analysis showed no clear association between LPI and postoperative peak cTnI when ACC time was ≤90 minutes, whereas longer intervals appeared more relevant when ACC time was >90 minutes. These findings suggest that the apparent effect of LPI was largely driven by operative complexity and ischaemic duration, and that single-dose blood-enriched modified del Nido cardioplegia may provide adequate myocardial protection within a 90-minute cross-clamp window.

This interpretation is supported by baseline and intraoperative differences across LPI groups. Patients with longer perfusion intervals also had longer ACC and CPB times and more repeat perfusions, indicating greater procedural complexity. In simplified models, adjustment for ACC time markedly attenuated the association between LPI and postoperative cTnI, whereas adjustment for the number of perfusions alone did not. These findings suggest that ACC duration was the main factor underlying the observed association between longer perfusion interval and greater myocardial injury.

At the same time, the impact of LPI varied by operative context. In patients with ACC time >90 minutes, the association with postoperative cTnI was stronger than in shorter ACC cases. Although the interaction was attenuated after multiple imputation and the missing-category sensitivity analysis, the consistent direction and larger point estimates suggest LPI may be more relevant under greater ischaemic burden, making the subgroup pattern biologically plausible and clinically informative.

Further support is that spline analysis in patients with ACC time >90 minutes showed a non-linear relationship between LPI and postoperative 48-hour peak cTnI, with a steeper rise beyond 80 minutes, suggesting a threshold effect where shorter intervals may be tolerated but vulnerability increases with prolonged ischaemia and perfusion. Given that this pattern was attenuated in sensitivity analysis, the 80-minute threshold should be considered exploratory and hypothesis-generating. Still, the similar direction across analyses supports the robustness of the complete-case findings.

Clinically, the protective adequacy of single-dose blood-enriched modified del Nido cardioplegia appears to depend on ischaemic duration. When ACC time was ≤90 minutes, perfusion interval was not clearly associated with higher postoperative peak cTnI, suggesting that a single perfusion of blood-enriched modified del Nido cardioplegia may be adequate within this ischaemic window. This interpretation is consistent with the reported 60-90 minute protection window for classical del Nido cardioplegia but should be viewed as supportive rather than definitive because perfusion strategy was not randomly assigned. In prolonged operations, longer intervals may warrant greater caution, although the apparent increase beyond 80 minutes remains exploratory. The absence of significant associations with adverse events likely reflects low event rates, limited end-point sensitivity, and other perioperative factors.

This study has several limitations. These include its retrospective design, potential residual confounding, close correlation between LPI and ACC duration, attenuation of interaction findings after multiple imputation, limited power for clinical end-points, lack of detailed perioperative factors and complications such as selective ultrafiltration, and the exploratory 80-minute threshold, which requires external validation.

## CONCLUSIONS

In video-assisted right infra-axillary MiMVS, the association between perfusion interval and postoperative myocardial injury depended on cross-clamp duration. Within an ACC duration of 90 minutes, single-dose blood-enriched modified del Nido cardioplegia appeared adequate, whereas longer intervals in prolonged procedures may warrant greater caution. The 80-minute threshold observed in prolonged cases remains exploratory. These findings support a context-dependent myocardial protection strategy.

## Supplementary Material

ivag174_Supplementary_Data

## Data Availability

Data underlying this article will be shared upon request to the corresponding authors.

## ABBREVIATIONS

ACC: Aortic cross-clamp
CI: Confidence interval
CPB: Cardiopulmonary bypass
cTnI: Cardiac troponin I
LPI: Longest perfusion interval
MiMVS: Minimally invasive mitral valve surgery
