# Supplementary material for: Perfusion Interval and Myocardial Injury in Minimally Invasive Mitral Valve Surgery: The Modifying Role of Aortic Cross-Clamp Duration
Source: Interdiscip Cardiovasc Thorac Surg. 2026 Jun 24;41(7):ivag174. doi: 10.1093/icvts/ivag174 (PMC13323797; doi:10.1093/icvts/ivag174)
Supplement: ivag174_Supplementary_Data [file ivag174_supplementary_data.docx]

Supplementary Table S1 Composition of Modified Del Nido cardioplegia

| Component | del Nido cardioplegia-A | del Nido cardioplegia-B |
| --- | --- | --- |
| Plasma-Lyte A(ml) | 500 | 500 |
| 20 % Mannitol(ml) | 8.15 | 8.15 |
| 25 % Magnesium Sulfate(ml) | 4 | 4 |
| 5 % Sodium Bicarbonate(ml) | 11.0 | 11.0 |
| 10 % Potassium Chloride(ml) | 20 | 10 |
| Lidocaine(ml) | 3.25 | 3.25 |

Supplementary Table S2 Extent of missing data for variables included in the analysis

| **Characteristic** | **Total N** | **Missing, n** | **Missing, %** |
| --- | --- | --- | --- |
| Age | 556 | 0 | 0 |
| Gender | 556 | 0 | 0 |
| Body mass index | 556 | 0 | 0 |
| History of coronary artery disease | 556 | 0 | 0 |
| History of diabetes mellitus | 556 | 0 | 0 |
| History of hypertension | 556 | 0 | 0 |
| Preoperative atrial fibrillation | 556 | 0 | 0 |
| Preoperative left ventricular ejection fraction | 556 | 15 | 2.70 |
| New York Heart Association classification | 556 | 246 | 44.24 |
| Preoperative cardiac troponin I | 556 | 0 | 0 |
| Cardiopulmonary bypass time | 556 | 0 | 0 |
| Aortic cross-clamp time | 556 | 0 | 0 |
| Number of perfusions | 556 | 0 | 0 |
| Longest perfusion interval | 556 | 0 | 0 |
| Blood transfusion | 556 | 0 | 0 |
| Postoperative peak cardiac troponin I within 48 h | 556 | 0 | 0 |
| Composite adverse events | 556 | 0 | 0 |

Supplementary Table S3 Univariable linear regression analyses for log₁₀-transformed postoperative peak cTnI within 48 h. Data are presented as β coefficient (95% confidence interval). The dependent variable was log_10_-transformed postoperative peak cTnI within 48 h. BMI to body mass index.

| Variable | β (95% CI) | P-value |
| --- | --- | --- |
| Aortic cross-clamp time (min) | 0.0126 (0.0096 to 0.0156) | <0.001 |
| Preoperative cardiac troponin I | 0.2024 (0.1538 to 0.2510) | <0.001 |
| Cardiopulmonary bypass time (min) | 0.0077 (0.0055 to 0.0099) | <0.001 |
| Number of perfusions | 0.5099 (0.3243 to 0.6955) | <0.001 |
| Longest perfusion interval (min) | 0.0169 (0.0103 to 0.0236) | <0.001 |
| Blood transfusion | 0.3911 (0.1747 to 0.6075) | <0.001 |
| Age (years) | 0.0111 (0.0044 to 0.0178) | 0.001 |
| Coronary artery disease | 0.3365 (0.1288 to 0.5442) | 0.002 |
| Hypertension | 0.2344 (0.0320 to 0.4368) | 0.023 |
| Preoperative atrial fibrillation | -0.1197 (-0.3598 to 0.1203) | 0.327 |
| Preoperative left ventricular ejection fraction (%) | 0.0042 (-0.0078 to 0.0162) | 0.491 |
| Preoperative cardiac function | -0.0427 (-0.1871 to 0.1018) | 0.561 |
| Diabetes mellitus | 0.0801 (-0.2045 to 0.3646) | 0.580 |
| Body mass index (kg/m²) | -0.0065 (-0.0343 to 0.0212) | 0.644 |
| Male sex | 0.0192 (-0.1824 to 0.2208) | 0.851 |

Supplementary Table S4 Multivariable linear regression analysis (Model 1) for log₁₀-transformed postoperative peak cTnI within 48 h. Data are presented as β coefficient (95% confidence interval). The dependent variable was log₁₀-transformed postoperative peak cTnI within 48 h. Model 1 was the core clinical model adjusted for longest perfusion interval to age to sex to body mass index to coronary artery disease to diabetes mellitus to hypertension to preoperative atrial fibrillation to preoperative cardiac function to preoperative left ventricular ejection fraction to preoperative cardiac troponin I and blood transfusion. BMI to body mass index.

| Variable | β (95% CI) | P-value |
| --- | --- | --- |
| Longest perfusion interval (min) | 0.0097 (0.0035 to 0.0159) | 0.002 |
| Age (years) | 0.0050 (-0.0018 to 0.0119) | 0.150 |
| Male sex | 0.0044 (-0.1753 to 0.1841) | 0.962 |
| Body mass index (kg/m²) | -0.0070 (-0.0318 to 0.0179) | 0.583 |
| Coronary artery disease | 0.2009 (0.0038 to 0.3981) | 0.046 |
| Diabetes mellitus | -0.0611 (-0.3146 to 0.1925) | 0.636 |
| Hypertension | 0.2653 (0.0778 to 0.4528) | 0.006 |
| Preoperative atrial fibrillation | -0.1846 (-0.4084 to 0.0392) | 0.106 |
| Preoperative cardiac function | -0.0456 (-0.1734 to 0.0822) | 0.483 |
| Preoperative left ventricular ejection fraction (%) | 0.0009 (-0.0099 to 0.0116) | 0.873 |
| Preoperative cardiac troponin I | 0.1808 (0.1325 to 0.2292) | <0.001 |
| Blood transfusion | 0.3361 (0.1372 to 0.5349) | <0.001 |
| N | 304 |  |
| Adjusted R² | 0.273 |  |

Supplementary Table S5 Multivariable linear regression analysis (Model 2) for log₁₀-transformed postoperative peak cTnI within 48 h. Data are presented as β coefficient (95% confidence interval). The dependent variable was log₁₀-transformed postoperative peak cTnI within 48 h. Model 2 was additionally adjusted for intraoperative complexity variables, including cardiopulmonary bypass time to aortic cross-clamp time and number of perfusions. BMI: body mass index; CI: confidence interval.

| Variable | β (95% CI) | P-value |
| --- | --- | --- |
| Longest perfusion interval (min) | -0.0006 (-0.0109 to 0.0097) | 0.913 |
| Age (years) | 0.0071 (0.0005 to 0.0137) | 0.035 |
| Male sex | -0.0117 (-0.1848 to 0.1615) | 0.895 |
| Body mass index (kg/m²) | -0.0096 (-0.0335 to 0.0143) | 0.429 |
| Coronary artery disease | 0.1853 (-0.0038 to 0.3743) | 0.055 |
| Diabetes mellitus | 0.0029 (-0.2416 to 0.2474) | 0.981 |
| Hypertension | 0.1957 (0.0141 to 0.3773) | 0.035 |
| Preoperative atrial fibrillation | -0.3185 (-0.5401 to -0.0970) | 0.005 |
| Preoperative cardiac function | -0.0374 (-0.1603 to 0.0855) | 0.550 |
| Preoperative left ventricular ejection fraction (%) | 0.0000 (-0.0105 to 0.0106) | 0.999 |
| Preoperative cardiac troponin I | 0.1469 (0.0987 to 0.1950) | <0.001 |
| Blood transfusion | 0.2498 (0.0552 to 0.4444) | 0.012 |
| Cardiopulmonary bypass time (min) | 0.0012 (-0.0023 to 0.0047) | 0.495 |
| Aortic cross-clamp time (min) | 0.0099 (0.0013 to 0.0185) | 0.024 |
| Number of perfusions | -0.1138 (-0.5184 to 0.2907) | 0.580 |
| N | 304 |  |
| Adjusted R² | 0.333 |  |

Supplementary Table S6 Collinearity diagnostics for multivariable Model 2. Data are presented as variance inflation factor. Model 2 included cardiopulmonary bypass time to aortic cross-clamp time and number of perfusions as intraoperative complexity variables. BMI to body mass index; VIF to variance inflation factor.

| Variable | VIF |
| --- | --- |
| Longest perfusion interval (min) | 3.352 |
| Age (years) | 1.407 |
| Sex | 1.109 |
| Body mass index (kg/m²) | 1.111 |
| Coronary artery disease | 1.205 |
| Diabetes mellitus | 1.108 |
| Hypertension | 1.190 |
| Preoperative atrial fibrillation | 1.276 |
| Preoperative cardiac function | 1.086 |
| Preoperative left ventricular ejection fraction (%) | 1.165 |
| Preoperative cardiac troponin I | 1.206 |
| Blood transfusion | 1.166 |
| Cardiopulmonary bypass time (min) | 3.221 |
| Aortic cross-clamp time (min) | 9.974 |
| Number of perfusions | 6.513 |
| *VIF: variance inflation factor.* | |

Supplementary Table S7 Comparison of simplified multivariable models for longest perfusion interval: complete-case versus multiple imputation analysis. Data are presented as β coefficient (95% CI) to unless otherwise indicated. The dependent variable was log₁₀-transformed postoperative peak cTnI within 48 h. Model 0 was the core clinical model. Model A was additionally adjusted for aortic cross-clamp time; Model B for number of perfusions; and Model C for both aortic cross-clamp time and number of perfusions. Missing data were handled using multiple imputation, and estimates were pooled across imputed datasets using Rubin’s rules. CI: confidence interval.

| Model | Complete-case analysis | | Multiple imputation analysis | |
| --- | --- | --- | --- | --- |
|  | β (95% CI) | P-value | β (95% CI) | P-value |
| Model 0: core clinical model | 0.0097 (0.0035 to 0.0159) | 0.002 | 0.0068 (0.0019 to 0.0116) | 0.006 |
| Model A: Model 0 + aortic cross-clamp time | 0.0016 (-0.0050 to 0.0082) | 0.635 | 0.0008 (-0.0045 to 0.0060) | 0.773 |
| Model B: Model 0 + number of perfusions | 0.0112 (0.0052 to 0.0173) | <0.001 | 0.0076 (0.0028 to 0.0124) | 0.002 |
| Model C: Model 0 + aortic cross-clamp time + number of perfusions | -0.0004 (-0.0107 to 0.0099) | 0.942 | -0.0045 (-0.0125 to 0.0035) | 0.270 |

Supplementary Table S8A Full multivariable linear regression results for simplified Model A in complete-case. Data are presented as β coefficient (95% confidence interval) to unless otherwise indicated. The dependent variable was log₁₀-transformed postoperative peak cTnI within 48 h. Model A was the core clinical model additionally adjusted for aortic cross-clamp time. CI: confidence interval.

| Variable | β (95% CI) | P-value |
| --- | --- | --- |
| Longest perfusion interval (min) | 0.0016 (-0.0050 to 0.0082) | 0.635 |
| Age (years) | 0.0071 (0.0005 to 0.0137) | 0.034 |
| Male sex | -0.0121 (-0.1839 to 0.1597) | 0.890 |
| Body mass index (kg/m²) | -0.0090 (-0.0328 to 0.0148) | 0.456 |
| Coronary artery disease | 0.1867 (-0.0018 to 0.3752) | 0.052 |
| Diabetes mellitus | 0.0043 (-0.2393 to 0.2478) | 0.973 |
| Hypertension | 0.1952 (0.0141 to 0.3762) | 0.035 |
| Preoperative atrial fibrillation | -0.3175 (-0.5370 to -0.0981) | 0.005 |
| Preoperative cardiac function | -0.0360 (-0.1582 to 0.0862) | 0.562 |
| Preoperative left ventricular ejection fraction (%) | -0.0009 (-0.0111 to 0.0094) | 0.867 |
| Preoperative cardiac troponin I | 0.1468 (0.0989 to 0.1947) | <0.001 |
| Blood transfusion | 0.2569 (0.0646 to 0.4492) | 0.009 |
| Aortic cross-clamp time (min) | 0.0092 (0.0058 to 0.0126) | <0.001 |
| N | 304 |  |
| Adjusted R² | 0.3360 |  |

Supplementary Table S8B Full multivariable linear regression results for simplified Model B in complete-case. Data are presented as β coefficient (95% confidence interval) to unless otherwise indicated. The dependent variable was log₁₀-transformed postoperative peak cTnI within 48 h. Model B was the core clinical model additionally adjusted for number of perfusions. CI: confidence interval.

| Variable | β (95% CI) | P-value |
| --- | --- | --- |
| Longest perfusion interval (min) | 0.0112 (0.0052 to 0.0173) | <0.001 |
| Age (years) | 0.0068 (0.0001 to 0.0134) | 0.047 |
| Male sex | -0.0238 (-0.1981 to 0.1505) | 0.788 |
| Body mass index (kg/m²) | -0.0074 (-0.0315 to 0.0166) | 0.543 |
| Coronary artery disease | 0.1972 (0.0064 to 0.3879) | 0.043 |
| Diabetes mellitus | 0.0030 (-0.2439 to 0.2499) | 0.981 |
| Hypertension | 0.2036 (0.0202 to 0.3869) | 0.030 |
| Preoperative atrial fibrillation | -0.3085 (-0.5315 to -0.0854) | 0.007 |
| Preoperative cardiac function | -0.0435 (-0.1672 to 0.0802) | 0.489 |
| Preoperative left ventricular ejection fraction (%) | -0.0008 (-0.0112 to 0.0096) | 0.874 |
| Preoperative cardiac troponin I | 0.1513 (0.1028 to 0.1998) | <0.001 |
| Blood transfusion | 0.2669 (0.0722 to 0.4616) | 0.007 |
| Number of perfusions | 0.4027 (0.2291 to 0.5763) | <0.001 |
| N | 304 |  |
| Adjusted R² | 0.3190 |  |

Supplementary Table S8C Full multivariable linear regression results for simplified Model C in complete-case. Data are presented as β coefficient (95% confidence interval) to unless otherwise indicated. The dependent variable was log₁₀-transformed postoperative peak cTnI within 48 h. Model C was the core clinical model additionally adjusted for aortic cross-clamp time and number of perfusions. CI: confidence interval.

| Variable | β (95% CI) | P-value |
| --- | --- | --- |
| Longest perfusion interval (min) | -0.0004 (-0.0107 to 0.0099) | 0.942 |
| Age (years) | 0.0071 (0.0005 to 0.0137) | 0.035 |
| Male sex | -0.0082 (-0.1810 to 0.1645) | 0.925 |
| Body mass index (kg/m²) | -0.0093 (-0.0331 to 0.0145) | 0.444 |
| Coronary artery disease | 0.1849 (-0.0041 to 0.3738) | 0.055 |
| Diabetes mellitus | 0.0010 (-0.2432 to 0.2452) | 0.994 |
| Hypertension | 0.1969 (0.0155 to 0.3783) | 0.034 |
| Preoperative atrial fibrillation | -0.3125 (-0.5332 to -0.0919) | 0.006 |
| Preoperative cardiac function | -0.0347 (-0.1571 to 0.0878) | 0.578 |
| Preoperative left ventricular ejection fraction (%) | -0.0008 (-0.0111 to 0.0095) | 0.881 |
| Preoperative cardiac troponin I | 0.1476 (0.0995 to 0.1956) | <0.001 |
| Blood transfusion | 0.2587 (0.0661 to 0.4514) | 0.009 |
| Aortic cross-clamp time (min) | 0.0110 (0.0031 to 0.0190) | 0.007 |
| Number of perfusions | -0.1016 (-0.5042 to 0.3011) | 0.620 |
| N | 304 |  |
| Adjusted R² | 0.3340 |  |

Supplementary Table S9 Collinearity diagnostics for simplified models assessing the association between longest perfusion interval and surgical complexity–related variables in complete-case. The table presents variance inflation factors (VIF) for longest perfusion interval, aortic cross-clamp time, and number of perfusions across three simplified models. Model A was the core clinical model additionally adjusted for aortic cross-clamp time; Model B for number of perfusions; and Model C for both aortic cross-clamp time and number of perfusions. Elevated VIF values in Model C indicate substantial multicollinearity among these variables. VIF: variance inflation factor.

| Model | Variable | VIF |
| --- | --- | --- |
| Model A | Longest perfusion interval (min) | 1.392991 |
| Model A | Aortic cross-clamp time (min) | 1.561127 |
| Model B | Longest perfusion interval (min) | 1.126240 |
| Model B | Number of perfusions | 1.175799 |
| Model C | Longest perfusion interval (min) | 3.342033 |
| Model C | Aortic cross-clamp time (min) | 8.581020 |
| Model C | Number of perfusions | 6.462993 |
| *Data are presented as variance inflation factor.* | | |

Supplementary Table S10 Multivariable linear regression model with interaction term for the association between longest perfusion interval and log₁₀-transformed postoperative peak cTnI within 48 h: complete-case versus multiple imputation analysis. β coefficients represent the change in log₁₀-transformed postoperative peak cTnI within 48 h per unit increase in each variable. The model included an interaction term between longest perfusion interval and aortic cross-clamp time (≤90 vs >90 min). All variables listed in the table were entered simultaneously into the multivariable linear regression model. Missing data were handled using multiple imputation, and estimates were pooled across imputed datasets using Rubin’s rules. ACC: aortic cross-clamp; BMI: body mass index; cTnI: cardiac troponin I.

| **Variable** | **Complete-case analysis** | | **Multiple imputation analysis** | |
| --- | --- | --- | --- | --- |
|  | **β (95% CI)** | **P value** | **β (95% CI)** | **P value** |
| Demographics |  |  |  |  |
| Age (per 1-year increase) | 0.008 (0.001 to 0.014) | 0.020 | 0.004 (-0.002 to 0.009) | 0.168 |
| Male sex | -0.016 (-0.189 to 0.156) | 0.851 | -0.026 (-0.160 to 0.109) | 0.708 |
| BMI | -0.009 (-0.033 to 0.015) | 0.460 | -0.001 (-0.020 to 0.019) | 0.950 |
| Clinical characteristics |  |  |  |  |
| Coronary artery disease | 0.214 (0.025 to 0.403) | 0.027 | 0.281 (0.122 to 0.440) | <0.001 |
| Diabetes | 0.018 (-0.226 to 0.262) | 0.886 | 0.148 (-0.080 to 0.376) | 0.201 |
| Hypertension | 0.168 (-0.015 to 0.350) | 0.071 | 0.189 (0.042 to 0.335) | 0.012 |
| Preoperative atrial fibrillation | -0.318 (-0.538 to -0.098) | 0.005 | -0.168 (-0.341 to 0.005) | 0.058 |
| Preoperative status |  |  |  |  |
| Cardiac function | -0.036 (-0.159 to 0.086) | 0.558 | -0.410 (-1.269 to 0.448) | 0.290 |
| Left ventricular ejection fraction | 0.001 (-0.009 to 0.011) | 0.854 | -0.436 (-1.268 to 0.396) | 0.252 |
| Preoperative cTnI | 0.140 (0.091 to 0.189) | <0.001 | -0.407 (-1.219 to 0.404) | 0.277 |
| Intraoperative factors |  |  | -0.001 (-0.010 to 0.008) | 0.796 |
| Longest perfusion interval (per 1-min increase) | 0.002 (-0.006 to 0.010) | 0.657 | 0.208 (0.165 to 0.251) | <0.001 |
| ACC time >90 min | -1.181 (-2.454 to 0.091) | 0.069 |  |  |
| Longest perfusion interval × cross-clamp time | 0.017 (0.003 to 0.032) | 0.018 | 0.003 (-0.004 to 0.009) | 0.435 |
| Blood product transfusion | 0.265 (0.072 to 0.457) | 0.007 | -0.015 (-1.053 to 1.023) | 0.977 |
| Number of perfusions | 0.381 (0.070 to 0.692) | 0.017 | 0.004 (-0.008 to 0.016) | 0.512 |
| Male sex was compared with female as the reference. Cross-clamp time >90 min was compared with ≤90 min. Blood product transfusion was coded as yes vs no. | | | | |

Supplementary Table S11 Subgroup analysis of the association between longest perfusion interval and log₁₀-transformed postoperative peak cTnI within 48 h according to cross-clamp time (≤90 vs >90 min). Data are presented as β coefficients (95% CI). Multiple imputation analysis: primary analysis using multiple imputed datasets for missing values. Sensitivity analysis: secondary analysis using a missing-category approach, where missing preoperative NYHA class values were treated as an “Unknown” category while retaining original ejection fraction values. Model 1 was adjusted for demographic and clinical variables (age, sex, body mass index, coronary artery disease, diabetes, hypertension, preoperative atrial fibrillation, cardiac function, ejection fraction, preoperative cTnI and blood transfusion). Model 2 was additionally adjusted for number of perfusions. P for interaction was obtained by including an interaction term between perfusion interval and cross-clamp time. ACC: aortic cross-clamp; CI: confidence interval.

|  | **Subgroup** | **Model** | **β (95% CI)** | **P-value** |
| --- | --- | --- | --- | --- |
| **Multiple imputation analysis** | ACC >90 min | Model 1 | 0.006 (-0.004, 0.015) | 0.219 |
|  | ACC >90 min | Model 2 | 0.010 (-0.004, 0.024) | 0.167 |
|  | ACC ≤90 min | Model 1 | 0.002 (-0.004, 0.008) | 0.567 |
|  | ACC ≤90 min | Model 2 | 0.002 (-0.004, 0.008) | 0.544 |
| **Sensitivity analysis** | ACC >90 min | Model 1 | 0.002 (-0.002, 0.006) | 0.329 |
|  | ACC >90 min | Model 2 | 0.005 (-0.002, 0.011) | 0.140 |
|  | ACC ≤90 min | Model 1 | 0.001 (-0.002, 0.003) | 0.625 |
|  | ACC ≤90 min | Model 2 | 0.001 (-0.002, 0.003) | 0.600 |

S

Supplementary Table S12 Restricted cubic spline ANOVA results and coefficients of covariates in the fully adjusted model. The table presents ANOVA results for the restricted cubic spline model, including both the overall and non-linear components of the longest perfusion interval. The table also shows the β coefficients (95% CI) and P values for covariates in the fully adjusted model, which was adjusted for age, sex, body mass index, coronary artery disease, diabetes, hypertension, preoperative atrial fibrillation, preoperative cardiac function, left ventricular ejection fraction, preoperative cTnI, blood product transfusion, and number of perfusions. cTnI: cardiac troponin I; CI: confidence interval.

| Section | Variable | β (95% CI) | P value |
| --- | --- | --- | --- |
| RCS ANOVA | Longest perfusion interval (overall) |  | <0.001 |
| RCS ANOVA | Non-linear component |  | 0.027 |
| Full model | Age | 0.007 (0.001 to 0.014) | 0.028 |
| Full model | Sex | 0.000 (-0.173 to 0.173) | 0.999 |
| Full model | Body mass index | -0.008 (-0.032 to 0.016) | 0.506 |
| Full model | Coronary artery disease | 0.208 (0.019 to 0.396) | 0.031 |
| Full model | Diabetes | 0.024 (-0.220 to 0.269) | 0.845 |
| Full model | Hypertension | 0.173 (-0.009 to 0.356) | 0.063 |
| Full model | Preoperative atrial fibrillation | -0.335 (-0.556 to -0.114) | 0.003 |
| Full model | Preoperative Cardiac function | -0.034 (-0.156 to 0.088) | 0.584 |
| Full model | Preoperative left ventricular ejection fraction | 0.000 (-0.010 to 0.011) | 0.935 |
| Full model | Preoperative cTnI | 0.138 (0.089 to 0.187) | <0.001 |
| Full model | Blood product transfusion | 0.282 (0.089 to 0.474) | 0.004 |
| Full model | Number of perfusions | 0.456 (0.277 to 0.635) | <0.001 |

Supplementary Table S13 Sensitivity analyses of the association between perfusion interval and log₁₀-transformed postoperative peak cTnI within 48 h in patients with cross-clamp time >90 min. Data are presented as β coefficient (95% confidence interval). Model 1 was the core clinical model adjusted for age to sex to body mass index to coronary artery disease to diabetes mellitus to hypertension to preoperative atrial fibrillation to preoperative cardiac function to preoperative left ventricular ejection fraction to preoperative cardiac troponin I and blood transfusion. Model 2 was additionally adjusted for the total number of perfusions. Model 2 was not fitted for count-based exposure variables due to collinearity with the total number of perfusions. CI: confidence interval.

| Exposure | Model | β (95% CI) | P value |
| --- | --- | --- | --- |
| Mean perfusion interval | Model 1 (n=187) | 0.007 (-0.002 to 0.015) | 0.114 |
|  | Model 2 (n=187) | 0.027 (0.013 to 0.042) | <0.001 |
| Number of intervals >80 min | Model 1 (n=187) | 0.209 (-0.111 to 0.529) | 0.199 |
| Proportion of intervals >80 min | Model 1 (n=187) | 0.088 (-0.305 to 0.481) | 0.657 |
|  | Model 2 (n=187) | 0.586 (-0.132 to 1.304) | 0.109 |
| Proportion of intervals >90 min | Model 1 (n=187) | -0.016 (-0.411 to 0.379) | 0.937 |
|  | Model 2 (n=187) | 0.306 (-0.508 to 1.121) | 0.458 |
| Number of intervals >90 min | Model 1 (n=187) | 0.082 (-0.270 to 0.435) | 0.644 |

Supplementary Table S14 Multivariable logistic regression analysis for the association between perfusion interval parameters and the secondary outcome. The secondary outcome was a composite of adverse events to defined as the occurrence of any of the following: postoperative mortality to extracorporeal membrane oxygenation (ECMO) support to intra-aortic balloon pump (IABP) support to continuous renal replacement therapy (CRRT) to permanent pacemaker implantation or postoperative cerebrovascular events.
The table presents odds ratios (OR) with 95% confidence intervals (CI) and P values for the associations between perfusion interval parameters (mean interval, maximum interval, and proportion of intervals >90 min) and the secondary outcome in the overall cohort and the >90 min subgroup. Two models were constructed: Model 1 was adjusted for age, sex, body mass index, coronary artery disease, diabetes mellitus, hypertension, preoperative atrial fibrillation, preoperative cardiac function, left ventricular ejection fraction, preoperative cardiac troponin I, and blood transfusion; Model 2 was additionally adjusted for the number of perfusions. OR: odds ratio; CI: confidence interval.

| Exposure | Cohort | Model 1  OR (95% CI) | P value | Model 2  OR (95% CI) | P value  (M2) |
| --- | --- | --- | --- | --- | --- |
| Mean interval | Overall | 0.999 (0.969 to 1.030) | 0.93 | 0.998 (0.968 to 1.030) | 0.89 |
|  | >90 min | 1.024 (0.973 to 1.086) | 0.36 | 1.021 (0.901 to 1.153) | 0.72 |
| Max interval | Overall | 0.993 (0.957 to 1.029) | 0.71 | 0.993 (0.957 to 1.029) | 0.70 |
|  | >90 min | 1.030 (0.966 to 1.112) | 0.36 | 1.023 (0.915 to 1.147) | 0.68 |
| Ratio >90 min | Overall | 2.889 (0.704 to 10.329) | 0.13 | 2.957 (0.696 to 11.281) | 0.14 |
|  | >90 min | 2.788 (0.367 to 29.105) | 0.30 | 2.938 (0.039 to 274.823) | 0.59 |
